# Efficacy, safety, and tolerability of once‐daily abediterol in patients with stable, persistent asthma: a Phase II, randomized, 7‐day, crossover study

**DOI:** 10.1002/prp2.356

**Published:** 2017-09-27

**Authors:** Jutta Beier, Rainard Fuhr, Beatriz Seoane, Eric Massana, Gonzalo de Miquel, Helena Pujol, Sandrine Ruiz

**Affiliations:** ^1^ insaf Respiratory Research Institute Biebricher Allee 34 65187 Wiesbaden Germany; ^2^ PAREXEL International GmbH Klinikum Westend, Haus 17 D‐14050 Berlin Germany; ^3^ AstraZeneca Avda. Diagonal 615 08028 Barcelona Spain; ^4^ Almirall Research and Development Centre Laureà Miró 408‐410 08980 Sant Feliu de Llobregat, Barcelona Spain; ^5^ Former employee of AstraZeneca Avda. Diagonal 615 08028 Barcelona Spain

**Keywords:** Asthma, bronchodilation, chronic respiratory disease, LABA

## Abstract

Abediterol is a once‐daily, long‐acting *β*_2_‐adrenergic agonist in development for the treatment of asthma and chronic obstructive pulmonary disease. We assessed the efficacy, safety, and tolerability of three dose levels of abediterol, given once daily for 7 days in patients with stable, persistent asthma. This was an ascending‐dose, three‐period incomplete crossover study design investigating three dose levels of abediterol versus placebo (EudraCT No. 2008‐003732‐38). Twenty‐eight male patients (25–59 years) were randomized to one of four treatment sequences (1:1:1:1). Follow‐up was 7 days after final treatment. Spirometry was performed regularly up to 24 h postdose Day 1, up to 36 h postdose Day 7, and at follow‐up. Vital signs, 12‐lead electrocardiogram, and clinical laboratory tests were recorded throughout. Abediterol 2.5, 5, and 10 *μ*g provided clinically and statistically significant improvements from baseline (predose, Day 1) in trough forced expiratory volume in 1 sec (FEV_1_) versus placebo on Day 7 (primary endpoint) of 334, 365, and 294 mL, respectively (all *P *<* *0.01), and peak FEV_1_ versus placebo on Day 7 of 364 (*P *<* *0.001), 403 (*P *<* *0.001), and 375 mL (*P *<* *0.01), respectively. Days 1 and 7 area under the curve (AUC) parameters within each abediterol group were similar for AUC_0–6_, AUC_0–12_, AUC_0–24_, and AUC_12–24_, with dose‐dependent effects observed on Day 1. Abediterol (2.5–10 *μ*g) demonstrated a good safety and tolerability profile. Abediterol 2.5, 5, and 10 *μ*g once daily achieved statistically and clinically significant improvements in pulmonary function versus placebo over 7 days and demonstrated a safety and tolerability profile comparable with placebo.

**Abbreviations**
AE: adverse event
ANCOVA: analysis of covariance
AUC: area under the curve
BMI: body mass index
CI: confidence interval
COPD: chronic obstructive pulmonary disease
ECG: electrocardiogram
FEF_25‐75_: forced mid‐expiratory flow
FEV_1_: forced expiratory volume in 1 sec
FVC: forced vital capacity
ICS: inhaled corticosteroids
LABA: long‐acting *β*_2_‐adrenergic agonist
LSM: least squares mean
PEF: peak expiratory flow
PT: preferred term
SAE: serious adverse event
SD: standard deviation
SE: standard error
TEAE: treatment‐emergent adverse event

## Introduction

Inhaled bronchodilators in the form of long‐acting *β*_2_‐adrenergic agonists (LABAs) in combination with an anti‐inflammatory agent, i.e. inhaled corticosteroids (ICS), are recommended for the treatment of patients with asthma that is uncontrolled by medium doses of corticosteroid monotherapy (Global Initiative for Asthma 2017). A number of LABA/ICS combinations are approved for the treatment of asthma, including fixed combinations of salmeterol/fluticasone propionate and formoterol/budesonide (Electronic Medicines Compendium 2016a, 2017). More recently, the first once‐daily fixed‐dose LABA/ICS combination of vilanterol/fluticasone furoate became available for the treatment of asthma and chronic obstructive pulmonary disease (COPD) (Electronic Medicines Compendium 2016b). In continuing efforts to tackle the burden of asthma at a personal as well as societal level, it remains necessary to examine ways of optimizing treatment outcomes. Once‐daily treatments may therefore play a role in this regard by potentially simplifying treatment regimens and aiding patient adherence to treatment (Cazzola and Matera 2008).

Abediterol napadisylate is a novel, once‐daily LABA in development as a fixed‐dose combination with an ICS or another anti‐inflammatory agent for the treatment of asthma and COPD (Aparici et al. 2012). Preclinical data show that abediterol is a potent, selective, and full *β*_2_‐adrenoceptor agonist with a rapid onset of action and long‐lasting effects (Aparici et al. 2012). Early‐phase clinical studies have shown that abediterol ≤10 *μ*g is well tolerated with no clinically relevant effects on heart rate, blood glucose, or serum potassium in healthy subjects, patients with asthma, or patients with COPD (Singh et al. 2014; Timmer et al. 2014; Beier et al. 2016). In patients with mild‐to‐moderate asthma, abediterol (5–25 *μ*g) demonstrated significant improvements in lung function compared with placebo and salmeterol 50 *μ*g twice daily as early as 5 min postdose, with a safety and tolerability profile consistent with that expected for a *β*_2_‐agonist (Beier et al. 2014). In another single‐dose study, abediterol 0.313–2.5 *μ*g provided rapid, dose‐dependent, clinically and statistically significant bronchodilation versus placebo that was maintained over 24 h in patients with asthma; a peak effect similar to salbutamol was also demonstrated, along with a good safety and tolerability profile (Singh et al. 2014).

In this study, we investigated the efficacy, safety, and tolerability of three doses of abediterol administered via Cyclohaler^®^ (Pharmachemie B.V., Haarlem, The Netherlands) once daily over 7 days in patients with stable, mild‐to‐moderate, persistent asthma.

## Materials and Methods

Male patients aged 18–70 years with a clinical diagnosis of persistent asthma (according to Global Initiative for Asthma guidelines (Global Initiative for Asthma 2017)) for ≥6 months prior to screening were eligible for inclusion in the study. Patients received a stable dose of ICS together with a short‐acting *β*_2_‐agonist or a LABA during the 6 weeks prior to screening and had not been exposed to any investigational medicinal product during that time. Patients were required to have a forced expiratory volume in 1 sec (FEV_1_) >60% and ≤85% of the predicted normal value, with an FEV_1_ reversibility ≥12% and an absolute increase in FEV_1_ of ≥200 mL over baseline value following inhalation of salbutamol 400 *μ*g at screening.

Patients were excluded from the study if they had a smoking history during the past 12 months or history of smoking greater than 10 pack‐years; presence or history of relevant clinically significant disease; hospitalization or emergency room treatment for acute asthma ≤6 weeks prescreening, between screening, and the first treatment period or between treatment periods; and required treatment with *β*_2_‐adrenergic antagonists.

The use of oral *β*_2_‐agonists, oral or inhaled anticholinergics, oral corticosteroids, theophylline or other xanthines (including slow release), leukotriene antagonists, nonselective *β*_1_‐blocking agents (selective *β*_1_‐blocking agents were permitted if stable ≥4 weeks prior to screening), non‐potassium‐sparing diuretics, antiarrhythmic agents, antidepressants, antipsychotics, macrolide antibiotics, fluoroquinolones, and antiprotozoal agents and other investigational drugs were prohibited during the study. Salbutamol pressurized metered dose inhaler (100 *μ*g/puff) was the only reliever medication permitted and was used on an as‐needed basis and avoided 6 h prior to and during a treatment visit unless absolutely necessary. Inhaled LABAs were permitted between treatment periods providing the dose remained fixed and were withheld from 72 h prior to study treatment until the last assessment of the respective treatment period.

### Compliance with study design

This was a Phase IIa, randomized, double‐blind, multiple‐dose, placebo‐controlled, three‐period, incomplete crossover, sequential, dose‐ascending study in male patients with persistent, mild‐to‐moderate asthma (EudraCT No. 2008‐003732‐38). The study was conducted at two sites in Germany. The study protocol, the patient's information, and consent form were approved by the Ethik‐Kommission bei der Landesärztekammer Hessen (an Independent Ethics Committee at the Chamber of Physicians, Hessen) and complied with the Declaration of Helsinki and the International Conference on Harmonisation and Good Clinical Practice guidelines. Patients provided written informed consent.

After screening, during which usual asthma treatment was withdrawn except for ICS and salbutamol rescue therapy, patients were randomized to one of four treatment sequences in a 1:1:1:1 ratio. During the study period, patients received three of the four treatments tested (abediterol 2.5, 5, or 10 *μ*g or placebo, administered once daily), each within a separate 7‐day treatment period, with each treatment period being separated by a 7‐ to 21‐day washout period. A follow‐up evaluation was performed 7 days after inhalation of the last treatment. Abediterol and placebo were administered in the morning as dry powder delivered from hard capsules inhaled through a rechargeable device (Cyclohaler^®^).

### Efficacy assessments

Pulmonary function tests (spirometry) were conducted at screening, during each treatment period, i.e., on Day 1 at predose, 5, 15, 30 min, then at 1, 2, 3, 4, 6, 8, 12, 14, 23, and 24 h postdose; on Day 4 at predose, and on Day 7 at the same time points as Day 1 (but not at 5 and 15 min) plus at 36 h postdose, and at follow‐up. Spirometers were used to measure FEV_1_, forced vital capacity (FVC), peak expiratory flow (PEF), and forced mid‐expiratory flow (FEF_25‐75_) and met American Thoracic Society and European Respiratory Society recommendations for accuracy and precision. To ensure stability of disease, the predose FEV_1_ on Day 1 of each treatment period was to be within 80–120% of the FEV_1_ measured at screening prior to salbutamol inhalation.

### Safety and tolerability assessments

Adverse events (AEs), including serious AEs (SAEs), were recorded throughout the study and follow‐up period. A complete physical examination was performed at screening and at follow‐up. Vital signs (blood pressure, pulse rate) and 12‐lead electrocardiogram (ECG) measures were made at screening, predose, and at 10 (15 min for the ECG) and 30 min, 1, 2, 4, 6, 8, 12, and 24 h postdose on Day 1 and Day 7 (with an additional time point at 36 h), as well as predose and 30 min postdose on Day 4, and at follow–up. Clinical laboratory tests (hematology, blood chemistry, urinalysis, and serology) were performed at screening, at 24 h postdose on Days 1 and 7, and at follow‐up. Coagulation tests were conducted at screening only. Serum potassium and blood glucose were determined at screening as well as at predose and 15 min, then at 4, 12, and 24 h postdose on Day 1 and Day 7 (with an additional time point at 36 h), and predose and 30 min after treatment on Day 4.

### Statistical analysis requirements

The primary efficacy endpoint was the least squares mean (LSM) change from baseline (predose Day 1) in trough FEV_1_ on Day 7 (LSM of the two highest FEV_1_ readings measured at 23 and 24 h after the final dose was administered). Secondary efficacy endpoints included: LSM change from baseline in trough FEV_1_ on Day 1, trough FVC, PEF, and FEF_25‐75_, and peak FEV_1_ on Days 1 and 7; time to peak FEV_1_ on Day 1; LSM change from baseline in normalized FEV_1_ and FVC area under the curve (AUC) 0–6, 0–12, 12–24, and 0–24 h postdose (Days 1 and 7) and 0–36 h (Day 7); and LSM change from baseline in FEV_1_, FVC, PEF, and FEF_25‐75_ at each time point on Days 1 and 7.

Analyses of all efficacy variables were performed on the per protocol population, defined as a subset of the safety population constituted by those patients who satisfied the main inclusion/exclusion criteria liable to affect the efficacy assessments, completed all treatment periods, and did not present serious violations of the protocol. All safety outcomes were analyzed using the safety population, defined as all randomized patients who received at least one dose of study medication.

The primary efficacy variable was analyzed using an analysis of covariance (ANCOVA) model for crossover designs; secondary efficacy variables were analyzed, according to the nature of variable, by means of ANCOVA models or descriptive statistics. Between‐treatments comparisons were performed by means of contrasts on the treatment factor. The difference between treatments was estimated by the difference between the LSM, its standard errors, and 95% confidence intervals. All statistical tests were performed using a 0.05, two‐sided significance level. All safety and tolerability data, number of withdrawals, and concomitant medications were analyzed by means of the appropriate descriptive statistics across treatment groups. All statistical analyses were performed using Statistical Analysis Software (SAS Institute Inc., Cary, NC, USA) version 8.2.

## Results

**Figure 1:**
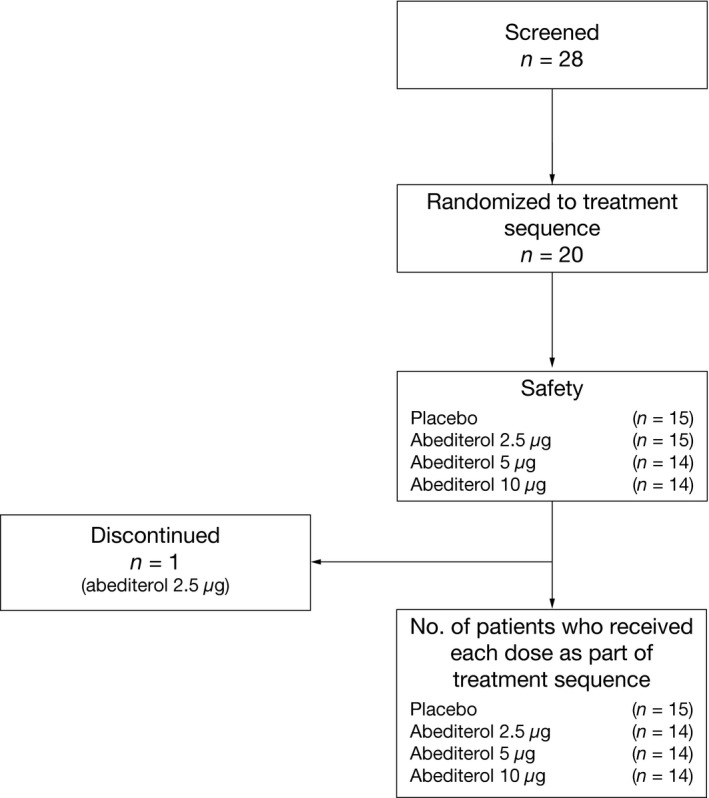
Flow diagram of the patients receiving one of four study treatment sequences. From 28 patients screened, 20 male patients with asthma who fulfilled the inclusion and exclusion criteria were included in the study. Patients were randomly assigned to one of the four possible treatment sequences, according to a randomization schedule in a 1:1:1:1 ratio. Each patient received three of the four treatments tested, each within a separate treatment period (7 days). One patient was withdrawn from the study at the end of Treatment Period 1 due to safety reasons and protocol compliance.

Of 28 male patients screened, 20 patients with stable, persistent asthma were randomized to treatment (mean age 42.7 ± 10.2 years). All patients were included in the safety population (Fig. 1). In total, 19 patients (per protocol population) completed the study and one patient withdrew due to an AE (acute bronchitis). Patient demographics are presented in Table 1.

**Table 1:** Patient demographics and baseline characteristics

Characteristic	Patients (*N* = 20)
Age (years), mean (SD)	42.7 (10.2)
Male, *n* (%)	20 (100.0)
White/Caucasian, *n* (%)	20 (100.0)
BMI (kg/m^2^), mean (SD)	26.5 (3.0)
Asthma duration (years), mean (SD)	21.7 (11.5)
Pre‐bronchodilation FEV_1_ (L), mean (SD)	2.9 (0.3)
FEV_1_ (% predicted), mean (SD)	73.2 (6.3)
Bronchial reversibility test (%), mean (SD)	22.7 (7.5)

BMI, body mass index; FEV_1_, forced expiratory volume in 1 sec; *N*, number of patients in the safety population (all patients who received at least one study dose); SD, standard deviation.

**Figure 2:**
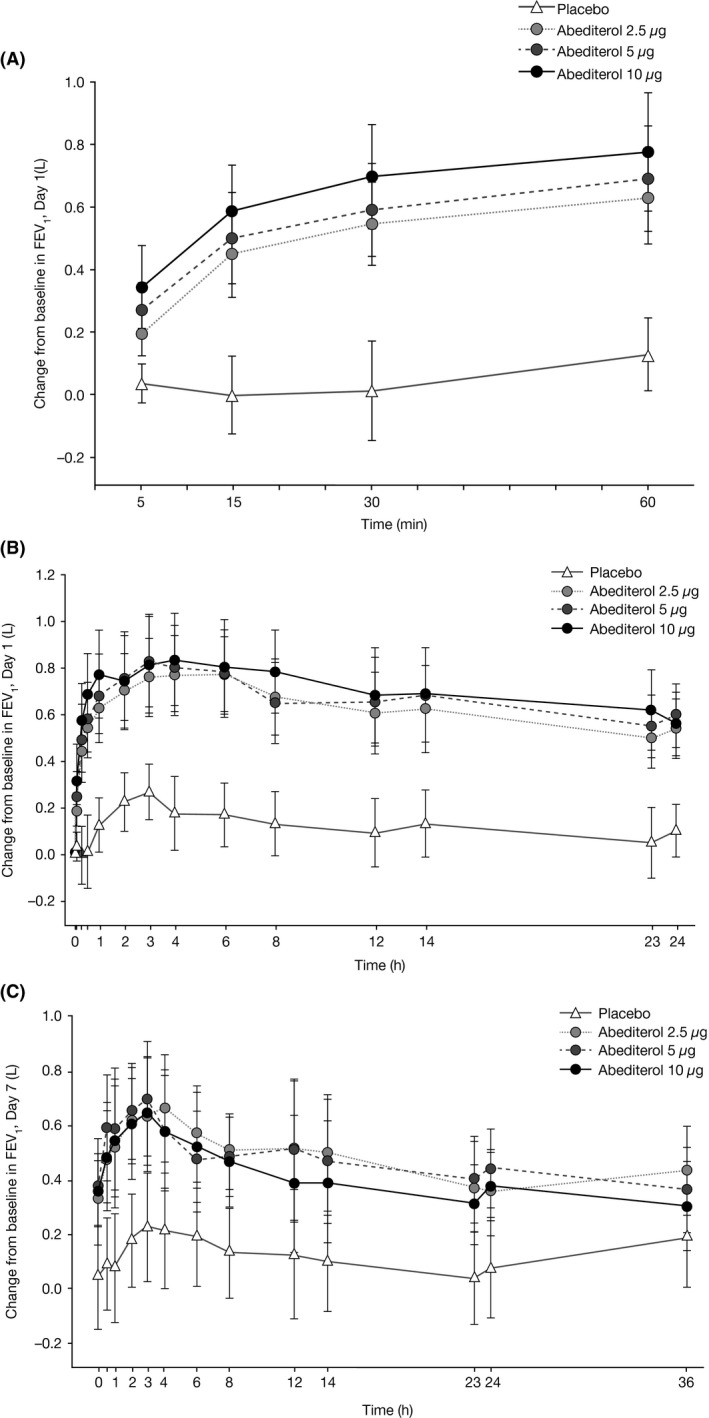
Change from baseline in FEV_1_ over time on (A) Day 1 (0–60 min), (B) Day 1 (0–24 h), and (C) Day 7 (0–36 h). The effect versus time profile for FEV_1_ absolute values shows that the bronchodilatory effect of all abediterol doses (2.5 *µ*g, 5 *µ*g, and 10 *µ*g) compared with placebo treatment was rapid (A) and sustained until the end of the 24‐h observation period on Day 1 (B) and until the end of the 36‐h period on Day 7 (C). All changes from baseline in FEV_1_ with abediterol compared with placebo were statistically significant at all time points on Day 7 (*P* < 0.05, except for the 36‐h time point with abediterol 10 *μ*g, *P* = 0.1871) and Day 1 (*P* < 0.001, except *P* < 0.01 for the abediterol 2.5 *µ*g dose at 5 min). FEV_1_, forced expiratory volume in 1 sec.

**Figure 3:**
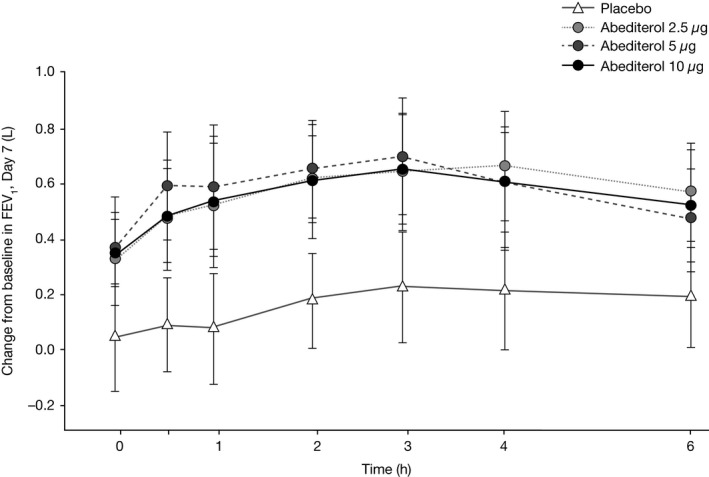
Change from baseline in FEV_1_ over time, Day 7, 0–6 h. The maximum adjusted mean change from baseline in FEV_1_ after administration of abediterol 2.5–10 *μ*g was observed within 2–4 h postdose on Day 7. There was also a slight increase from baseline in FEV_1_ after administration of placebo, which peaked at 3 h postdose. FEV_1_, forced expiratory volume in 1 sec.

All doses of abediterol significantly increased trough FEV_1_ from baseline (predose Day 1) compared with placebo on Day 7 (adjusted mean change 0.334, 0.365, and 0.294 L with abediterol 2.5, 5, and 10 *μ*g, respectively; all *P *<* *0.01; Table 2). On Day 1, the increase in trough FEV_1_ from baseline (predose Day 1) with abediterol (all doses) compared with placebo was greater than on Day 7 (adjusted mean change 0.444, 0.500, and 0.520 L with abediterol 2.5, 5, and 10 *μ*g, respectively; all *P *<* *0.0001; Table 2). No dose–response relationship was evident for the effect of abediterol on change from baseline in trough FEV_1_ at Day 1 or Day 7. Significant improvements in FEV_1_ with abediterol (all doses) compared with placebo were observed from 5 min postdose on Day 1 (Fig. 2A) and these changes were sustained until the end of the 24‐h period on Day 1 (Fig. 2B) and up to 36 h on Day 7 (Fig. 2C). Changes from baseline in FEV_1_ with abediterol compared with placebo were considered clinically relevant and statistically significant at all time points on Day 7 (*P *<* *0.05; except for the 36‐h time point with abediterol 10 *μ*g, *P *=* *0.1871) (Fig. 2C) and Day 1 (*P *<* *0.001; except for the 5‐min time point with abediterol 2.5 *μ*g, *P *<* *0.01) (Fig. 2B). At Day 7, the majority of patients reached their peak FEV_1_ within 2–4 h postdose (Fig. 3). Change from baseline in peak FEV_1_ was also clinically and significantly greater with abediterol compared with placebo on Day 1 and Day 7; adjusted mean change following abediterol 2.5, 5, and 10 *μ*g versus placebo was 0.482, 0.548, and 0.565 L, respectively, on Day 1 (all *P *<* *0.0001), and 0.364, 0.403, and 0.375 L on Day 7 (all *P *<* *0.01; Table 2).

**Table 2:** Change from baseline (LSM difference vs. placebo) in trough and peak FEV_1_ on Day 1 and Day 7 with abediterol

Baseline	Mean FEV_1,_ L (SD)		
Abediterol 2.5 *μ*g	2.875 (0.352)		
Abediterol 5 *μ*g	2.886 (0.536)		
Abediterol 10 *μ*g	2.881 (0.410)		
	LSM difference vs. placebo (SE)	95% CI	*P*‐value
Change from baseline in trough FEV_1_ on Day 1, L			
Abediterol 2.5 *μ*g	0.444 (0.084)	0.267, 0.621	<0.0001
Abediterol 5 *μ*g	0.500 (0.089)	0.311, 0.688	<0.0001
Abediterol 10 *μ*g	0.520 (0.098)	0.313, 0.728	<0.0001
Change from baseline in trough FEV_1_ on Day 7, L			
Abediterol 2.5 *μ*g	0.334 (0.095)	0.132, 0.536	0.0029
Abediterol 5 *μ*g	0.365 (0.097)	0.158, 0.571	0.0018
Abediterol 10 *μ*g	0.294 (0.100)	0.081, 0.506	0.0098
Change from baseline in peak FEV_1_ on Day 1, L			
Abediterol 2.5 *μ*g	0.482 (0.063)	0.349, 0.615	<0.0001
Abediterol 5 *μ*g	0.548 (0.069)	0.402, 0.693	<0.0001
Abediterol 10 *μ*g	0.565 (0.082)	0.391, 0.739	<0.0001
Change from baseline in peak FEV_1_ on Day 7, L			
Abediterol 2.5 *μ*g	0.364 (0.072)	0.211, 0.517	0.0001
Abediterol 5 *μ*g	0.403 (0.084)	0.224, 0.582	0.0002
Abediterol 10 *μ*g	0.375 (0.101)	0.160, 0.590	0.0020

Baseline = predose measurement on Day 1.

CI, confidence interval; FEV_1_, forced expiratory volume in 1 sec; LSM, least squares mean; SD, standard deviation; SE, standard error.

On both Day 1 and Day 7, normalized AUC FEV_1_ parameters within each abediterol dose group were similar for AUC_0–6_, AUC_0–12_, AUC_0–24_, and AUC_12–24_ (Table 3). AUC FEV_1_‐adjusted means on Day 1 showed dose‐dependent increases across all time intervals examined, ranging from 0.567 to 0.701 L for abediterol 2.5 *μ*g, 0.621 to 0.747 L for abediterol 5 *μ*g, and 0.658 to 0.774 L for abediterol 10 *μ*g. AUC FEV_1_‐adjusted means on Day 7 were not dose‐dependent and ranged from 0.454 to 0.593 L for abediterol 2.5 *μ*g, 0.451 to 0.586 L for abediterol 5 *μ*g, and 0.356 to 0.554 L for abediterol 10 *μ*g. Differences between AUC FEV_1_ values with abediterol versus placebo at Days 1 and 7 were considered clinically relevant and statistically significant (*P *<* *0.05).

**Table 3:** Change from baseline in normalized AUC for FEV_1_ on Days 1 and 7

	Placebo *N* = 15	Abediterol 2.5 *μ*g *N* = 14	Abediterol 5 *μ*g *N* = 14	Abediterol 10 *μ*g *N* = 14
*LSM (SE) and 95% CI*				
Change from baseline in AUC FEV_1_ on Day 1, L				
AUC_0–6_	0.176 (0.053)	0.701 (0.070)	0.747 (0.080)	0.774 (0.090)
	(0.064, 0.289)	(0.552, 0.851)	(0.577, 0.917)	(0.584, 0.964)
AUC_0–12_	0.150 (0.057)	0.683 (0.072)	0.711 (0.080)	0.759 (0.088)
	(0.029, 0.272)	(0.530, 0.835)	(0.541, 0.881)	(0.571, 0.946)
AUC_0‐24_	0.122 (0.054)	0.627 (0.069)	0.666 (0.076)	0.707 (0.082)
	(0.009, 0.236)	(0.481, 0.772)	(0.505, 0.828)	(0.534, 0.879)
AUC_12–24_	0.096 (0.055)	0.567 (0.067)	0.621 (0.073)	0.658 (0.078)
	(−0.021, 0.212)	(0.426, 0.708)	(0.466, 0.775)	(0.493, 0.823)
Change from baseline in AUC FEV_1_ on Day 7, L				
AUC_0–6_	0.171 (0.091)	0.593 (0.093)	0.586 (0.096)	0.554 (0.097)
	(−0.021, 0.363)	(0.395, 0.791)	(0.382, 0.789)	(0.348, 0.759)
AUC_0–12_	0.165 (0.086)	0.565 (0.090)	0.541 (0.092)	0.500 (0.092)
	(−0.017, 0.347)	(0.374, 0.757)	(0.345, 0.736)	(0.306, 0.695)
AUC_0–24_	0.125 (0.084)	0.517 (0.092)	0.500 (0.093)	0.428 (0.089)
	(−0.053, 0.302)	(0.322, 0.712)	(0.303, 0.697)	(0.239, 0.618)
AUC_12–24_	0.074 (0.080)	0.454 (0.090)	0.451 (0.092)	0.356 (0.087)
	(−0.095, 0.244)	(0.262, 0.645)	(0.257, 0.645)	(0.171, 0.540)
AUC_0–36_	0.129 (0.081)	0.484 (0.090)	0.459 (0.090)	0.390 (0.084)
	(−0.043, 0.301)	(0.293, 0.676)	(0.269, 0.650)	(0.212, 0.568)

Baseline = predose measurement on Day 1.

AUC, area under the curve; CI, confidence interval; FEV_1_, forced expiratory volume in 1 sec; LSM, least squares mean; *N*, number of patients in the per protocol population (patients who completed all treatment periods and presented no serious violation of the protocol); SE, standard error.

Similar clinically relevant and statistically significant increases from baseline with abediterol versus placebo were observed for PEF and FEF_25‐75_ on Days 1 and 7 (*P *<* *0.05 for all). Peak FEV_1_ values (the highest FEV_1_ value observed over the first 4 h) following abediterol treatments on Day 1 were reached at a median time of 180 min (3 h) postdose.

### Safety and tolerability

Single and multiple doses of abediterol 2.5, 5, and 10 *μ*g were generally safe and well tolerated in male patients with stable asthma. A dose–response relationship was observed after repeated dosing for some of the safety and tolerability outcomes measured, such as change in pulse rate, change in heart rate, and the occurrence of tremor and nervousness. Overall, 15 patients (75%) reported 56 treatment‐emergent AEs (TEAEs; 28 mild, 23 moderate, and five severe). The proportion of patients who experienced at least one TEAE was 40%, 57%, and 50% in the abediterol 2.5, 5, and 10 *μ*g groups, respectively, compared with 47% in the placebo group (Table 4). There was a dose‐dependent increase in the number of patients with tremors from zero patients treated with 2.5 *μ*g abediterol to two patients treated with 5 *μ*g abediterol and five patients treated with 10 *μ*g abediterol. Similarly, there was a dose‐dependent increase in the number of patients with nervousness from zero patients treated with 2.5 *μ*g and 5 *μ*g abediterol to three patients treated with 10 *μ*g abediterol. The most common TEAEs (Table 4) were headache (15 events in 11 patients) and tremor (eight events in seven patients). Five TEAEs were considered severe: concussion, skeletal injury, headache, and migraine (in two patients). These severe TEAEs were not considered related to the study medication in any treatment group. No SAEs or deaths occurred during the study. One patient withdrew due to bronchitis, which was moderate in severity, occurred several days after inhalation of abediterol 2.5 *μ*g, and was not related to study medication.

**Table 4:** Patients with ≥1 TEAE by PT and treatment group (safety population)

	Placebo (*N* = 15) *n* (%)	Abediterol 2.5 *μ*g (*N* = 15) *n* (%)	Abediterol 5 *μ*g (*N* = 14) *n* (%)	Abediterol 10 *μ*g (*N* = 14) *n* (%)	Total (*N* = 20) *n* (%)
Any event	7 (46.7)	6 (40.0)	8 (57.1)	7 (50.0)	15 (75.0)
Headache	4 (26.7)	4 (26.7)	0 (0.0)	3 (21.4)	7 (35.0)
Tremor	0 (0.0)	0 (0.0)	2 (14.3)	5 (35.7)	6 (30.0)
Dyspnea	1 (6.7)	3 (20.0)	1 (7.1)	0 (0.0)	4 (20.0)
Nervousness	0 (0.0)	0 (0.0)	0 (0.0)	3 (21.4)	3 (15.0)
Nasopharyngitis	1 (6.7)	1 (6.7)	0 (0.0)	0 (0.0)	2 (10.0)
Migraine	1 (6.7)	0 (0.0)	2 (14.3)	1 (7.1)	2 (10.0)
Ocular hyperemia	0 (0.0)	0 (0.0)	1 (7.1)	0 (0.0)	1 (5.0)
Bronchitis	0 (0.0)	1 (6.7)	0 (0.0)	0 (0.0)	1 (5.0)
Herpes zoster	0 (0.0)	0 (0.0)	1 (7.1)	0 (0.0)	1 (5.0)
Concussion	0 (0.0)	0 (0.0)	1 (7.1)	0 (0.0)	1 (5.0)
Skeletal injury	1 (6.7)	0 (0.0)	0 (0.0)	0 (0.0)	1 (5.0)
Back pain	1 (6.7)	0 (0.0)	0 (0.0)	0 (0.0)	1 (5.0)
Myalgia	0 (0.0)	1 (6.7)	0 (0.0)	0 (0.0)	1 (5.0)
Dizziness	0 (0.0)	1 (6.7)	0 (0.0)	1 (7.1)	1 (5.0)
Somnolence	0 (0.0)	0 (0.0)	1 (7.1)	0 (0.0)	1 (5.0)
Involuntary muscle contractions	0 (0.0)	1 (6.7)	0 (0.0)	0 (0.0)	1 (5.0)
Initial insomnia	0 (0.0)	0 (0.0)	0 (0.0)	1 (7.1)	1 (5.0)
Cough	1 (6.7)	0 (0.0)	0 (0.0)	0 (0.0)	1 (5.0)
Oropharyngeal pain	0 (0.0)	1 (6.7)	0 (0.0)	0 (0.0)	1 (5.0)

*N*, number of patients in the safety population; *n*, number of patients in the specific category; PT, preferred term; TEAE, treatment‐emergent adverse event.

There was no evidence of clinically meaningful effects on serum potassium or on blood glucose after inhalation of abediterol at any dose on Day 1 or 7. No clinically significant findings were found in the 12‐lead ECG parameters that were assessed. A small increase in heart rate from baseline was observed with abediterol compared with placebo on Day 7. This increase was dose‐dependent until 36 h postdose (except for the time point of 4 h) and reached a maximum value of 10.7 bpm at 6 h with abediterol 10 *μ*g. The maximum mean increases in heart rate in the other abediterol‐treated groups were 6.1 bpm at 36 h postdose with abediterol 2.5 *μ*g and 7.5 bpm at 8 h postdose with abediterol 5 *μ*g. In comparison, the mean increase in the placebo group reached a maximum of 4.1 bpm 36 h postdose. Overall, this increase was not considered to be clinically significant and as this was not designed as a QT study these data were only analyzed using descriptive statistics.

On Day 7, a slight numerical increase from baseline in QT corrected by Bazett's formula (QTcB; maximum mean increase from predose on Day 1, 14.9 msec with abediterol 10 *μ*g vs. 3.4 msec with placebo at 6 h postdose) and QT corrected by Fridericia's formula (QTcF; maximum mean increase from predose on Day 1, 6.93 msec with abediterol 10 *μ*g at 4 h postdose vs. 2.53 msec with placebo at 8 h postdose) was observed with abediterol compared with placebo. Similarly, normalized AUC_0–24_ QTcB and QTcF showed a slight numerical increase with abediterol versus placebo on Day 7 (normalized AUC_0–24_ QTcB, 403.05 msec with abediterol 10 *μ*g vs. 394.26 msec with placebo; normalized AUC_0–24_ QTcF, 395.10 msec with abediterol 10 *μ*g vs. 392.34 msec with placebo). However, as the trial was not specifically designed for a thorough QT evaluation, only descriptive statistics were performed and a cautious assessment of these data should be made.

## Discussion

In this study in patients with stable, persistent asthma, single and multiple doses of abediterol 2.5, 5, and 10 *μ*g provided a marked and sustained, clinically relevant and statistically significant improvement in lung function compared with placebo, with no dose‐dependent effects. Onset of action was rapid and maximum bronchodilation was observed at 2–4 h postdose.

Later dose‐ranging studies of abediterol in patients with stable asthma (dose range 0.313–2.5 *μ*g (Singh et al. 2014)) and in patients with stable COPD (dose range 0.625–10 *μ*g (Beier et al. 2016) once daily) have demonstrated clinically and statistically significant dose‐related improvements in bronchodilation compared with placebo. The lack of dose–response in bronchodilatory effect in this study suggests that the doses tested were at the top of the dose–response curve. This is further supported by an earlier study that examined a range of abediterol doses from 5 to 25 *μ*g in patients with stable asthma (Beier et al. 2014) where no dose–response relationship was observed.

The bronchodilator effects of abediterol (trough FEV_1_ vs. placebo) on Day 7, while still exceeding the predose by 0.294–0.365 L, were less pronounced than on Day 1, which may indicate some tachyphylaxis, and this was most evident with the highest dose compared with lower doses. This rapid reduction in bronchodilator pharmacodynamic response is widely acknowledged with the LABA class of drugs, however, and will be explored in subsequent studies using doses of abediterol in the therapeutic range. Overall, abediterol demonstrated a good safety and tolerability profile, comparable with other studies in the abediterol development program (Beier et al. 2014; Singh et al. 2014; Timmer et al. 2014); the nature of the reported events was consistent with the known safety and tolerability profile of LABAs (Decramer et al. 2013).

A dose–response relationship was observed after multiple doses of abediterol for some of the safety and tolerability assessments, including increases in heart rate. Such effects are expected with *β*_2_‐adrenergic agonist administration. The lowest dose of abediterol (2.5 *μ*g), inhaled once in the morning over 7 days, proved to be as safe and tolerable as placebo and showed a bronchodilator activity that was not distinguishable from the two higher doses of abediterol tested (5 and 10 *μ*g).

## Conclusion

In conclusion, in patients with stable, persistent asthma, once‐daily abediterol 2.5–10 *μ*g provided clinically relevant and statistically significant improvements from baseline in bronchodilation (including LSM trough and peak FEV_1_) compared with placebo over a 7‐day treatment period. In addition, abediterol 2.5–10 *μ*g showed a good overall safety and tolerability profile at all doses examined that was consistent with those of other *β*_2_‐agonists and similar to placebo at the lowest dose evaluated. No bronchodilatory dose–response effect was seen at the relatively high doses of abediterol examined; further studies of lower doses are required to measure/examine the lower end of the dose–response curve.

## Author Contributions

All authors contributed to the conception and design of the study, data analysis/interpretation and revision of the manuscript for intellectual content, and provided final approval of the manuscript. JB was the principal investigator of the study.

## Disclosure

Jutta Beier has received sponsorship to carry out studies, together with some consultancy fees and honoraria, from several pharmaceutical companies; these include Novartis, Almirall, Revotar, Cytos, Boehringer/Pfizer, Mundipharma, and Berlin Chemie. Eric Massana is an employee of Almirall. Beatriz Seoane, Sandrine Ruiz, and Helena Pujol are employees of AstraZeneca PLC. Gonzalo de Miquel is a former employee of AstraZeneca PLC. Rainard Fuhr is an employee of PAREXEL, a clinical research organization that was sponsored to conduct the study. Beatriz Seoane, Gonzalo de Miquel, and Sandrine Ruiz have a patent pending for abediterol (novel dosage form and formulation of abediterol, WO2013178742 A1).
